# The kynurenine pathway as a potential link between ethanol-induced behavioral alterations and neuroinflammation

**DOI:** 10.3389/fphar.2025.1628527

**Published:** 2025-07-07

**Authors:** Leticia Gil de Biedma-Elduayen, Pablo Giménez-Gómez, Nuria Morales-Puerto, Rebeca Vidal, Álvaro Del Río-García, Carlos Núñez-de la Calle, Lluna Careaga, María Dolores Gutiérrez-López, Esther O’Shea, María Isabel Colado

**Affiliations:** ^1^ Departamento de Farmacología y Toxicología, Facultad de Medicina, Universidad Complutense, Madrid, Spain; ^2^ Instituto de Investigación Sanitaria Hospital 12 de Octubre, Madrid, Spain; ^3^ Red de Investigación en Atención Primaria de Adicciones del Instituto de Salud Carlos III, Madrid, Spain; ^4^ Instituto Universitario de Investigación Neuroquímica (IUIN), Facultad de Medicina, Universidad Complutense, Madrid, Spain; ^5^ The Brudnick Neuropsychiatric Research Institute, Department of Neurobiology, University of Massachusetts Chan Medical School, Worcester, MA, United States

**Keywords:** ethanol, kynurenine, chronic intermittent ethanol (CIE), immune system, anxiety, memory

## Abstract

**Introduction:**

The neuroimmune actions of ethanol have recently gained significant attention. Concurrently, the kynurenine pathway, the main catabolic route of tryptophan (TRP), has emerged as a novel target for modulating drug abuse and as a critical immune regulator. This pathway is implicated in behavioral and cognitive alterations, including anxiety, depression, and memory impairment—conditions closely associated with ethanol (EtOH) dependence. The kynurenine pathway is activated under inflammatory and immune conditions.

**Objective:**

We previously demonstrated that chronic EtOH consumption increases kynurenine (KYN) levels in mice. Here, we investigate the effect of EtOH dependence and withdrawal on behavioral and cognitive parameters, the nucleus accumbens (NAc) transcriptome, and KYN, TRP and serotonin (5-HT) levels and KYN/TRP and 5-HT/TRP ratios in mice.

**Methods:**

Adult male mice were subjected the Chronic Intermittent ethanol (CIE) paradigm, a model for dependence and withdrawal. Twenty-four hours post-EtOH exposure, we analyzed behavioral and cognitive parameters, sequenced the NAc transcriptome, and measured KYN, TRP and 5-HT levels as well as KYN/TRP and 5-HT/TRP ratios in plasma, limbic forebrain, cortex and cerebellum using HPLC.

**Results:**

The CIE model induced anxiety-like behavior and memory impairment. Transcriptomic analysis of the NAc revealed immune system activation, including upregulation of immune and inflammation-related genes. Furthermore, chronic EtOH exposure increased KYN levels and the KYN/TRP ratio across plasma and brain regions.

**Conclusion:**

This study suggests that chronic EtOH exposure induces neuroimmune activation, which may trigger KYN pathway activation and contribute to anxiety and memory deficits observed in the CIE model.

## 1 Introduction

Ethanol (EtOH), commonly known as alcohol, is the most extensively consumed drug worldwide, contributing to approximately 5% of the global disease burden and being associated with over 200 illnesses and injuries (World Health Organization, 2019). The consumption of EtOH triggers oxidative stress and inflammation, processes that contribute to tissue damage and conditions such as liver injury, digestive and cardiovascular diseases, cognitive impairments, and neuropsychiatric disorders (Pohorecky and Brick, 1988; Hayes et al., 2016; McHugh and Weiss, 2019). Persistent and excessive EtOH intake can lead to dependence, a chronically relapsing disorder characterized by compulsive drug-seeking, impaired control over intake, and the development of a negative emotional state (e.g., dysphoria, anxiety, irritability) when access to EtOH is restricted (Koob, 2014). This condition involves adaptive changes in the brain’s reward and stress systems (Koob, 2014). To explore dependence and withdrawal, we used the chronic intermittent ethanol (CIE) model in mice. This well-established model incorporates repeated cycles of EtOH inhalation, voluntary consumption, and withdrawal, leading to an escalation in voluntary EtOH intake (Lopez and Becker, 2005; Lopez et al., 2017; Gil de Biedma-Elduayen et al., 2022).

The neuroimmune actions of EtOH have gained significant attention in recent years. It has been proposed that EtOH-induced inflammation in the central nervous system (CNS) may constitute a mechanism by which EtOH affects brain function. Proinflammatory signals are thought to disrupt behavioral control systems and the mesolimbic reward system, potentially contributing to excessive EtOH consumption in certain models (Coleman and Crews, 2018; Erickson et al., 2019).

Studies involving animal models and individuals diagnosed with alcohol use disorder (AUD) indicate that EtOH activates the immune system through various receptors, including those belonging to the toll-like receptor family, within microglia and astrocytes (Crews et al., 2013; Rubio-Araiz et al., 2017; Coleman and Crews, 2018). This is further corroborated by multiple transcriptomic studies, which reveal overexpression of genes associated with immune responses and inflammation in diverse EtOH exposure models across distinct brain regions (Osterndorff-Kahanek et al., 2013; Osterndorff-Kahanek et al., 2015; Zhang et al., 2015; Mccarthy et al., 2018; McClintick et al., 2018).

The kynurenine (KYN) pathway, the primary catabolic route of the tryptophan (TRP), has emerged as a novel target for modulating drug abuse (Justinova et al., 2013; Giménez-Gómez et al., 2018; Morales-Puerto et al., 2021; Gil de Biedma-Elduayen et al., 2022), as well as a critical regulator of immune function (O’Farrell and Harkin, 2017). Approximately 95% of TRP is metabolized into KYN through the action of two enzymes: tryptophan 2,3-dioxygenase (TDO), primarily located in the liver, and indolamine 2,3-dioxygenase (IDO), which is widely distributed across various tissues (Morales-Puerto et al., 2021). The remaining 5% of TRP is converted into serotonin (5-HT) by tryptophan hydroxylase (TPH). In the brain, KYN can be synthesized locally via IDO, though more than half of brain KYN is taken up from the periphery (Fukui et al., 1991).

Thus, under homeostatic conditions, TDO is the main enzyme responsible for KYN synthesis. However, during inflammatory events, the activity of IDO increases, leading to elevated extrahepatic KYN levels. In such circumstances, IDO plays a relevant role in regulating TRP metabolism within the brain (O’Farrell and Harkin, 2017; Kim and Jeon, 2018). Disruptions in the KYN pathway, particularly during inflammatory states, have been associated with neurodegenerative diseases, psychiatric conditions such as anxiety and depression, and cognitive impairments (O’Farrell and Harkin, 2017; Kim and Jeon, 2018; Chirico et al., 2020) - factors closely linked with EtOH abuse (Hayes et al., 2016; McHugh and Weiss, 2019). Previous clinical (Badawy et al., 2009; Gleissenthall et al., 2014; Neupane et al., 2015; Vidal et al., 2020) and preclinical research (Giménez-Gómez et al., 2019; Jiang et al., 2020; dos Santos et al., 2021), suggests that EtOH administration activates TDO and IDO enzymes, leading to KYN accumulation, an effect observed even during periods of abstinence.

In this study, we aimed to investigate the effects of the CIE dependence and withdrawal model in mice on behavioral and cognitive parameters, the transcriptomic profile of the nucleus accumbens (NAc) and on the levels of KYN, TRP and 5-HT in both the periphery and the brain.

## 2 Materials and methods

### 2.1 Animals

Eight-week-old male C57BL/6J mice (Envigo, United States) were used and maintained in conditions of constant temperature (21 °C± 2°C) and a 12-h reverse light cycle. For this study, we used a total of 136 male mice. Ninety-six mice were used for the behavioral tests: sixteen for each test (8 Control and 8 CIE). For the RNA sequencing and analysis, eight mice were used (4 Control and 4 CIE). Finally, we used 32 mice to measure KYN, TRP and 5-HT levels (24 and 72 h after the last EtOH exposure).

Mice were initially housed in groups of eight with *ad libitum* access to food and water for 5 days. Next, they were individually housed, randomly assigned to either the Control or CIE group, and habituated to drinking water *ad libitum* from a drinking pipette for 7 days.

### 2.2 Experimental design

The CIE paradigm, a validated EtOH dependence and relapse drinking model, was conducted as previously described (Lopez et al., 2017; Gil de Biedma-Elduayen et al., 2022; Olivencia et al., 2023). First, mice underwent a two-bottle choice limited access (2BC) procedure to establish baseline EtOH and water intake. For 2 weeks, they had 2-h access to two drinking pipettes containing either 15% v/v EtOH or water from Monday to Friday. Water and EtOH intake were measured daily, and EtOH preference was calculated as follows: 15% EtOH intake/total fluid intake x 100. The Control group had no access to EtOH at any time. Subsequently, the CIE group was exposed to EtOH vapor in inhalation chambers (CIE: 16 h/day for 4 days), whereas Control group inhaled air alone. Air flux and ethanol concentration in the inhalation chambers were adjusted daily to maintain a plasma EtOH concentration between 150–250 mg/dL (Lopez et al., 2017; Gil de Biedma-Elduayen et al., 2022).

**FIGURE 1:**
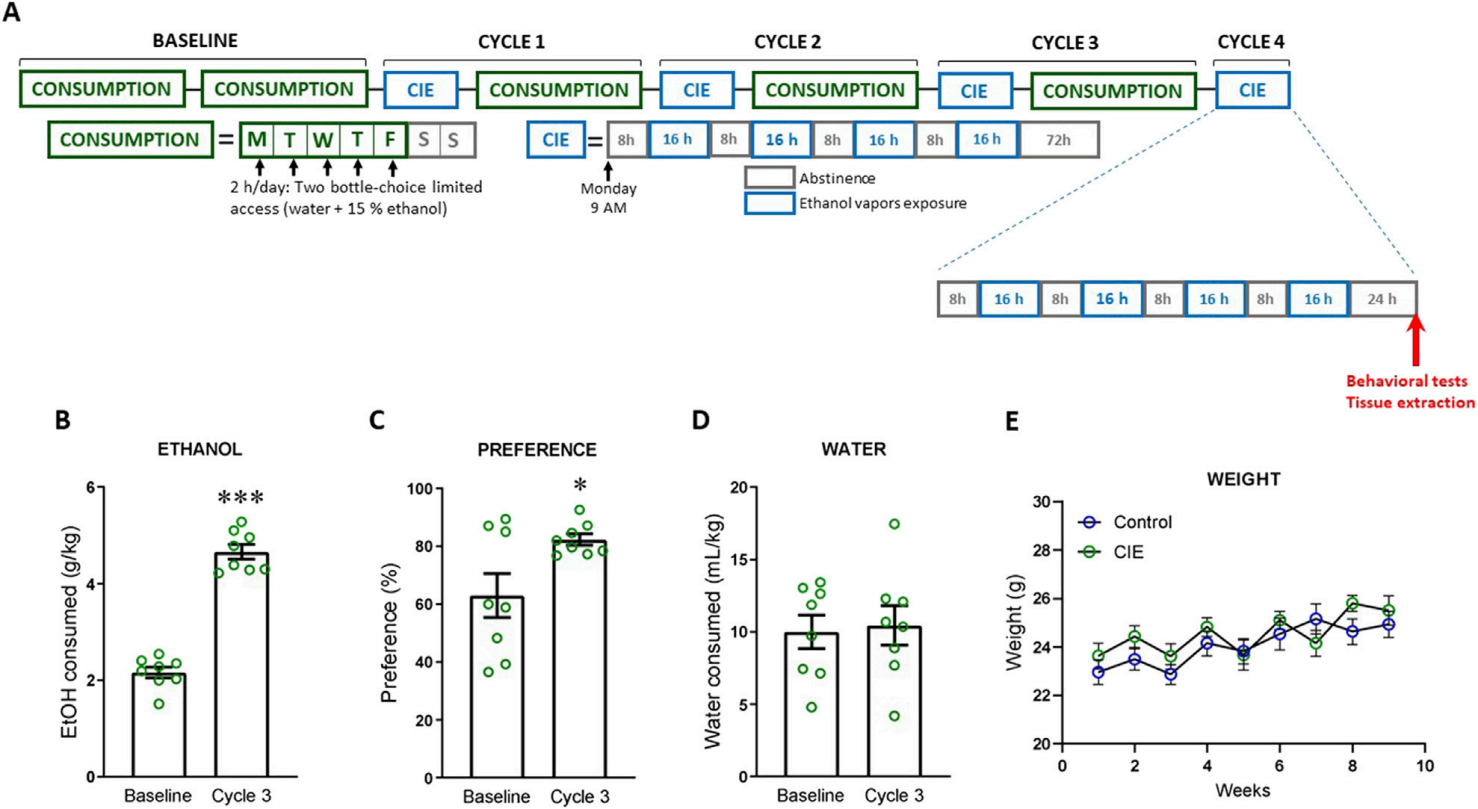
Chronic intermittent ethanol (CIE) vapor exposure paradigm. **(A)** Schematic timeline for CIE paradigm to induce dependence in mice. **(B–D)** Average EtOH consumption, preference for EtOH and water consumption, respectively, during the two-bottle choice limited access (2BC) procedure over baseline weeks and after three cycles of CIE vapor exposure. **(E)** Weight gain of mice exposed to EtOH vapor (CIE group) compared with those exposed to air (Control group) throughout the CIE paradigm. Results are presented as mean ± SEM; n = 8 for each group. Statistically different from baseline: *p < 0.05, ***p < 0.001, using *t*-test **(B–D)** and two-way ANOVA **(E)**.

Before being introduced in the chambers, mice were injected i. p. with 1.6 g/kg EtOH and 1 mmol/kg pyrazole, an alcohol dehydrogenase inhibitor (Lopez et al., 2017; Gil de Biedma-Elduayen et al., 2022). Control animals received saline and pyrazole. Following the fourth inhalation session, mice underwent a 72-h abstinence period, followed by 5 days of the same 2BC protocol used during baseline. This CIE + 2BC regimen was repeated for three cycles, with a fourth cycle consisting of only four inhalation sessions. Twenty-four or 72 hours after the last inhalation session, mice were subjected to various behavioral tests or euthanized by decapitation (Figure 1A).

Consistent with previous studies (Lopez et al., 2017; Gil de Biedma-Elduayen et al., 2022; Olivencia et al., 2023), the CIE paradigm produced an escalation in voluntary EtOH consumption (Figure 1B; t (14) = 13.43, p < 0.0001) and preference for EtOH (Figure 1C; t (14) = 2.45, p = 0.028), without altering water consumption (Figure 1D; t (14) = 0.25, p = 0.805). Weekly weight measurements showed no difference in weight gain between animals subjected to the CIE paradigm and the Control group (Figure 1E). Two-way ANOVA analysis revealed a significant time effect (F (8, 126) = 4.42, p < 0.001), but no effect of treatment (F (1, 126) = 3.67, p = 0.058) and no interaction between factors (F (8, 126) = 0.81, p = 0.597).

### 2.3 Behavioral tests

All behavioral tests were conducted 24 h after the last exposure to EtOH vapors (Figure 1A), following established protocols (Espejo-Porras et al., 2013; Ledesma et al., 2017; Giménez-Gómez et al., 2019). In addition, elevated plus maze (EPM) and novel object recognition (NOR) were also performed at 72 h post-EtOH exposure to explore if the behavioral changes persisted over time. Prior to beginning any behavioral testing, mice were transported to a dimly lit test room and allowed a 1-h habituation period. Different sets of animals were used for each behavioral test to avoid interference between them. All the behavioral tests were analyzed by a blinded observer.

To assess locomotor activity, the Open Field test (OFT) was conducted using a computer-assisted actimeter (Actitrack, Panlab, Spain). This apparatus consists of a 45 × 45 cm enclosure surrounded by infrared sensors positioned 1 cm from the ground and 2.5 cm apart. These are connected to a control unit that analyzes various locomotor parameters. The horizontal activity (distance travelled in cm) and the average speed of the mice were recorded over a 10-min period.

Anhedonia was assessed using the Sucrose Preference Test, based on a 2-h free-choice paradigm with two pipettes available for 8 h. One pipette contained a 1% sucrose solution while the other contained water. The positions of the pipettes were alternated between the cages to prevent potential place preferences. No food or water deprivation was carried out before conducting the test. Sucrose preference was calculated as the ratio of sucrose intake to total liquid intake. A decrease in sucrose preference is widely accepted in the literature as an indicator of anhedonia (Giménez-Gómez et al., 2019).

The EPM paradigm was performed to assess the anxiety levels of the animals. Mice were placed in the center of the EPM consisting of four arms (30 × 5 cm), two open and two enclosed, forming a central platform (5 × 5 cm) at the intersection point, elevated 45 cm above the floor. The animals were allowed 5 minutes to freely explore the maze. The number of entries into the open arms and the percentage of time spent in the open arms were used as indicators of anxiety (Ledesma et al., 2017).

Memory function was evaluated using the NOR test. Mice were placed in an open box (24 × 24 × 15 cm) initially containing two identical objects and allowed 3 minutes of free exploration. After this, they were returned to their home cage for a 1-min interval during which one object was replaced with a new and distinct object. The animals were then reintroduced to the box for another 3 minutes of exploration. The position of the objects in the cage was alternated between animals and experimental groups to minimize any potential place preference. Object exploration was defined as intentional contact with the object by the mouse’s snout or front paws within 2 cm of the object. The time spent exploring the old (familiar) and new (novel) objects during the second exposure was recorded, and the discrimination index (DI) was calculated using the formula: DI = (tnovel - tfamiliar)/(tnovel + tfamiliar) × 100 (Ledesma et al., 2017).

### 2.4 Sample extraction

Twenty-four hours after the final exposure to EtOH vapors, the mice were euthanized by decapitation (Figure 1A). Trunk blood was collected in 10 mL K_2_-EDTA tubes (BD, United States) and immediately centrifuged twice at 1300 x *g* (4°C) for 10 min to obtain plasma, which was then stored at −80°C. Brains were extracted and dissected on ice using a mouse brain matrix for coronal slices (World Precision Instruments, United States). After removing the olfactory bulbs, brain tissue anterior to the optic chiasm was collected and referred to as “limbic forebrain” (Giménez-Gómez et al., 2018; Giménez-Gómez et al., 2021; Gil de Biedma-Elduayen et al., 2022). The cortex of the remaining brain portion, as well as the cerebellum, were also collected. The transcriptomic analysis was conducted on the NAc. Using a matrix, 1 mm thick sections were made, and the NAc was dissected with a punch (Stoelting Co., United States), between bregma 1.98–0.62 mm. The distinct brain regions were either stored at −80°C or preserved in the appropriate buffer for subsequent use.

### 2.5 RNA sequencing and analysis

RNA extraction, sequencing and analysis were performed by Macrogen (Korea). RNA was extracted using the RNeasy Mini Kit from Qiagen following the manufacturer’s protocol. DNase treatment was applied to remove DNA contamination. The RNA integrity was verified using Agilent Technologies 2,100 bioanalyzer, with a RIN value ≥7. Paired-end libraries were then generated using the TruSeq Stranded mRNA Sample Prep Kit (Illumina^®^, United States). After PCR amplification, fragments with insert sizes between 200–400 base pairs were selected. The cDNA libraries were sequenced using sequencing by synthesis technology (Illumina^®^, United States) and the quality of reads was evaluated using FastQC v.0.11.7 (Babraham Bioinformatics, United Kingdom). To minimize biases, Trimmomatic 0.38 was used to remove low-quality reads, adapter sequences, contaminating DNA, and PCR duplicates (Usadel Lab, Germany).

cDNA fragments were mapped to the *Mus musculus* (mm10) genome using HISAT2 v.2.1.0 (Kim Lab, United States) and the Bowtie2 2.3.4.1 aligner (SourceForge, United States). Known genes and transcripts were assembled with StringTie v.2.1.3b (Johns Hopkins University, United States) based on the reference genome. The abundance of genes/transcripts was quantified in terms of read counts, FPKM (fragments per kilobase of transcript per million mapped reads) and TPM (transcripts per million per kilobase). The read count values of known genes obtained using the -e option of StringTie were used as the original raw data (45,777 genes). The analysis involved filtering low-quality transcripts, normalizing data, and removing samples with multiple zero read count. To minimize systematic bias, size factors were estimated using the “estimate Size Factors” method, and normalization was carried out using the Relative Log Expression (RLE) method in the DESeq2 R library. Log2-transformed values (read count+1) and regularized log(rlog) transformations were used for data visualization. Using the normalized values, similarity tests between samples were conducted, including correlation analysis, multidimensional scaling, and hierarchical clustering.

Differentially expressed genes between the Control and CIE groups were determined using Fold Change (FC) and the binomial Wald Test, performed via DESeq2 for pairwise comparisons. Genes with |FC| ≥ 2 and *P* < 0.05 were considered significant. This analysis provided a list of differentially expressed genes, which was then used for hierarchical clustering analysis and graphical representation via a heatmap and volcano plot. Additionally, a gene set enrichment analysis based on gene ontology was conducted using the g:Profiler tool (http://geneontology.org/) to identify prevalent molecular functions or signaling pathways within the set of differentially expressed genes. All data files are available upon request.

### 2.6 KYN, TRP, and 5-HT measurements in plasma and brain

Samples were processed and normalized to tissue weight as previously described (Giménez-Gómez et al., 2018; Giménez-Gómez et al., 2019; Giménez-Gómez et al., 2021; Abuin-Martínez et al., 2021; Gil de Biedma-Elduayen et al., 2022). Plasma samples were deproteinized by adding 75 μL of 6% perchloric acid to the mixture of 50 μL plasma and 125 μL of water. Limbic forebrain, cortex and cerebellum samples were homogenized in five volumes of deionized water by sonication (Labsonic 2000U, B. Braun Melsungen AG, Germany) at 30% amplitude for 15 s. The homogenates were deproteinized by adding 25 μL of 6% perchloric acid per 100 µL of sample. In both cases, the acidified samples were vortexed and kept at room temperature for 10 min before being centrifuged at 16,000 *g* for 15 min at 4°C to collect the supernatants.

For KYN measurement, 20 μL (plasma) or 60 μL (limbic forebrain, cortex and cerebellum) of supernatant were applied onto a reversed-phase column (80 mm × 4.6 mm, 3 mm; HR-80; Thermo Fisher Scientific, United States). KYN was isocratically eluted using a mobile phase containing 0.1 M sodium acetate and 4% acetonitrile, pH 4.6, at a flow rate of 1 mL/min. KYN was measured by UV detection at 360 nm using a 2487 UV detector (Waters, United States). Under these conditions, the retention time for KYN was 4.2 min. For TRP and 5-HT measurement, 20 μL of supernatant were applied to the same column, and TRP and 5-HT were isocratically eluted using a mobile phase containing 0.1 M ammonium acetate and 8% methanol, pH 3.8 (adjusted with glacial acetic acid), at a flow rate of 1 mL/min. TRP and 5-HT were detected fluorometrically at excitation/emission wavelengths of 270/360 nm and 290/398 nm, respectively using a 2,475, Multi Fluorescence Detector (Waters, United States). Under these conditions, retention times were 7 min for TRP and 4.5 min for 5-HT.

### 2.7 Data and statistical analyses

Initial group size (n = 8) was selected based on our previous experience to ensure a statistical significance considering the possible loss of mice due to the application of the criteria of humane endpoints if physical signs other than ethanol intoxication were observed, loss of consumption data due to malfunction of the pipette tip or outlier values (determined by the extreme studentized deviate method [ROUT test] with significance level of Q = 1%). These three conditions were the only exclusion criteria employed and account for differences in group sizes. For the RNA sequencing and analysis, the initial group size was n = 4, considering the high sensitivity and analytical power of this technique.

Data are presented as mean ± SEM. The threshold for statistical significance was set at *P* < 0.05. Normality was assessed using the Shapiro–Wilk test (Supplementary Table S1). Comparisons between two groups were conducted using an unpaired Student’s t-test when the data met the assumption of normality, and the Mann-Whitney *U* test when they did not. For analyses involving two independent variables, a two-way ANOVA was used. As the only two-way ANOVA performed (Figure 1E) did not show statistically significant main effects or interaction between factors, no *post hoc* multiple comparisons tests were conducted. All statistical analyses were performed using GraphPad Prism 8.0 (GraphPad Software Inc., United States). Sample extraction, processing, and data collection and analyses were carried out by a researcher blinded to the treatment group of each animal.

## 3 Results

### 3.1 The CIE model produces anxiety-like behavior and memory impairment

**FIGURE 2:**
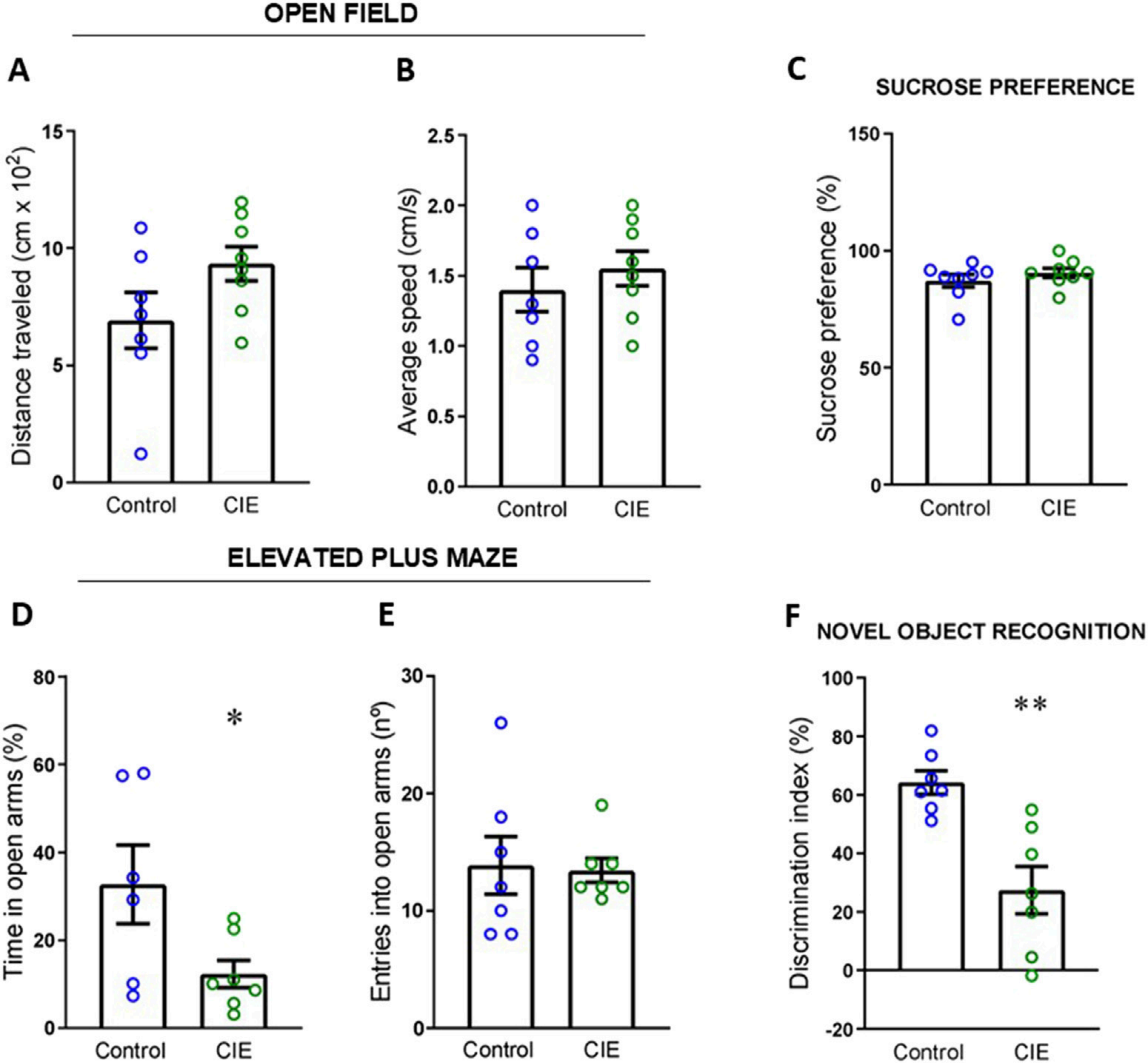
Effect of the CIE model on behavioral and cognitive parameters in dependent mice. **(A)** Distance travelled and, **(B)** average speed in the open-field test. **(C)** Sucrose preference (%) test. **(D)** Time spent in open arms (%) and, **(E)** number of entries into open arms in the elevated plus maze test. **(F)** Discrimination index (%) in the novel object recognition test. All the tests were conducted 24 h after the final exposure to EtOH vapors. Results are presented as mean ± SEM; n: Control = 7 and CIE = 8 for A and B; Control and CIE = 8 for C; Control and CIE = 7 for D, E and F Statistically different from Control: *p < 0.05, **p < 0.01, using *t*-test for A, B, D, F, and *U* test for C, E.

To assess the effect of CIE model exposure on the selected behavioral and cognitive parameters, the behavioral tests were performed 24 h after the last exposure to EtOH vapors. Analysis of locomotor activity via the OFT revealed no significant differences in the distance traveled (Figure 2A; t (13) = 1.79, p = 0.097) or average speed (Figure 2B; t (13) = 0.77, p = 0.457) between the Control and CIE groups. Similarly, no differences were found in sucrose preference, used as an indicator of anhedonia, between the two groups (Figure 2C; U = 24.5, p = 0.458, exact). Anxiety levels were assessed using the EPM, measuring the percentage of time spent in and the number of entries into the open arms as indicators. A reduction in the percentage of time spent in the open arms was observed in the CIE animals (Figure 2D; t (11) = 2.28, p = 0.043), while the number of entries did not change (Figure 2E; U = 21.5, p = 0.730, exact). This suggests that CIE animals entered the open arms as frequently as the Control animals but spent less time exploring them, indicating a higher level of anxiety in the CIE group. Finally, the NOR test was used to evaluate memory by assessing the DI between the new and old objects. The Student’s t*-*test revealed a decrease in the discrimination index within the CIE group (Figure 2F; t (12) = 4.04, p = 0.002), indicating memory impairment 24 h after vapor exposure.

To determine whether the observed anxiety- and memory-related changes persisted over time, we conducted EPM and NOR tests again at 72 h post-withdrawal. At this later time point, no significant differences were found between Control and CIE groups in the time spent in (Supplementary Figure S1A; t (14) = 0.726, p = 0.480) or entries into the open arms (Supplementary Figure S1B; t (14) = 0.214, p = 0.834) of the EPM. Similarly, no differences in the DI were observed in the NOR test (Supplementary Figure S1C; t (12) = 0.413, p = 0.687). These results suggest that the behavioral alterations identified at 24 h post-withdrawal did not persist at 72 h.

### 3.2 RNA sequencing suggests an activation of the immune system in the NAc produced by the CIE model

After describing the alterations in anxiety and memory produced by the CIE model, we performed RNA sequencing to examine gene expression profiles in the NAc of mice 24 h after the last vapor exposure. Post-sequencing, a total of 45,777 known genes were identified as raw, unprocessed data. Filtering out low-quality transcripts and normalizing the data resulted in 18,857 genes retained for statistical analysis, while 26,920 were excluded (Supplementary Figure S2A). Quality assessments demonstrated high sample similarity, as shown by correlation analysis based on Pearson coefficients (Supplementary Figure S1B), multidimensional scaling (Supplementary Figure S1C), and hierarchical clustering (Supplementary Figure S1D), with no outlier samples identified.

**FIGURE 3:**
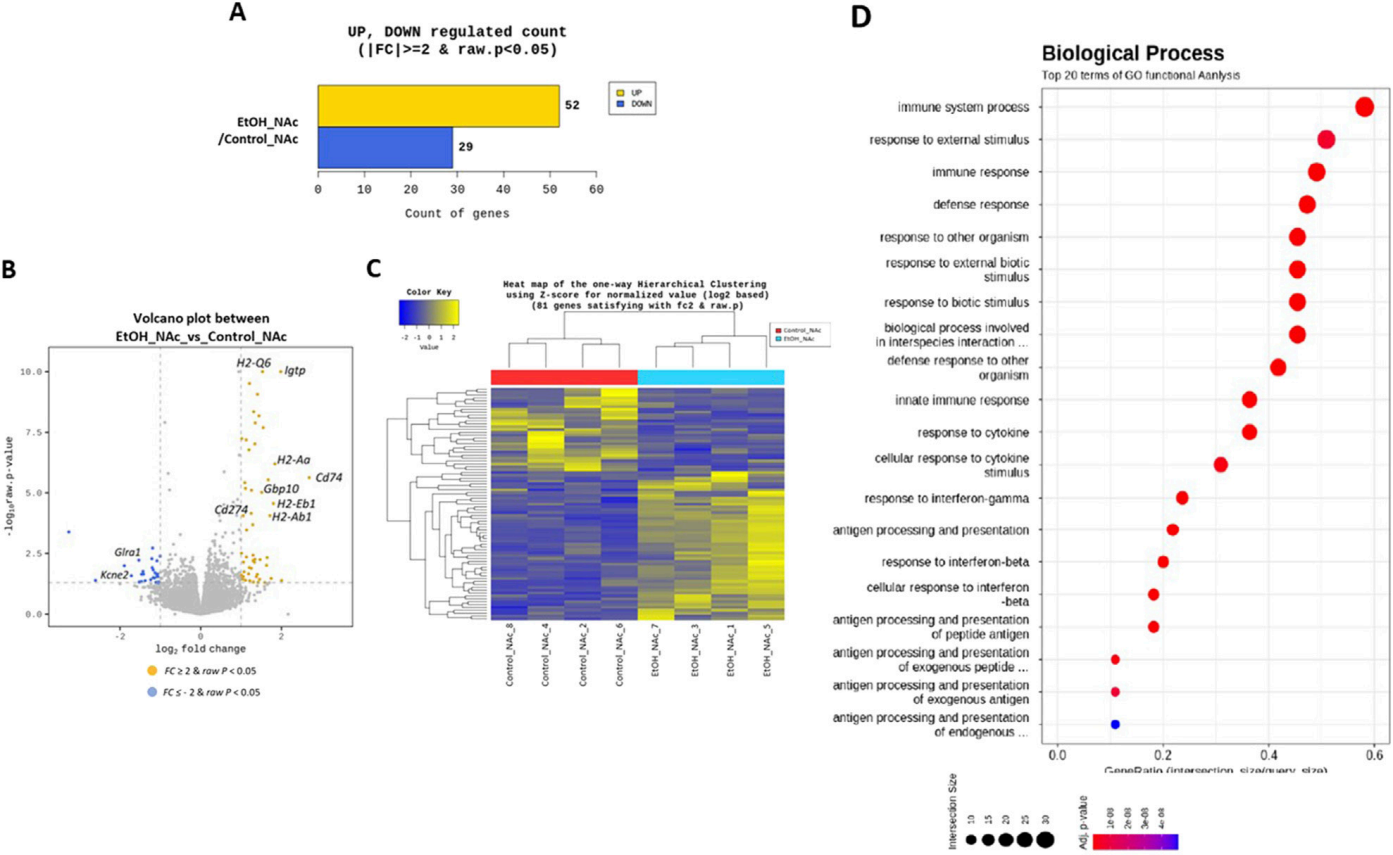
Transcriptomic profile in the nucleus accumbens (NAc) of mice exposed to the CIE model. **(A)** Count of upregulated and downregulated genes based on the Fold Change (ǀFCǀ ≥2) and the p-value (*P* < 0.05). The comparison pair was Control vs CIE using the statistical method FC and binomial Wald Test, n = 4. **(B)** Volcano plot presenting the differentially expressed genes. It shows the log2 of the FC and -log10 of the p-value obtained from the comparison between Control and CIE groups. Genes with upregulation (FC ≥ 2 and *P* < 0.05, in yellow) appear in the upper right, while genes with downregulation (FC ≤ −2 and *P* < 0.05, in blue) appear in the upper left. **(C)** A heatmap representing hierarchical clustering of differentially expressed genes. The genes are hierarchically grouped using Euclidean distance. On the X-axis, samples are ordered according to the group, and on the left side of the Y-axis, hierarchical clustering is performed associating genes based on their expression profile in different samples. Genes downregulated (FC < −2) are represented in blue, those upregulated (FC > 2) in yellow, while intermediate colors represent genes with ‘standard’ expression. **(D)** Main results of the enrichment analysis based on gene ontology. The dot plot displays the top 20 results of the ontology analysis concerning biological processes.

Statistical analysis identified 81 differentially expressed genes between the Control and CIE groups based on fold change (|FC| ≥ 2) and a significance threshold of *P* < 0.05 (Figures 3A,B; Supplementary Table S2). Among these genes, 52 were upregulated, and 29 were downregulated in the CIE group (Figure 3A). Hierarchical clustering affirmed strong correlations among samples within the same group (Figure 3C).

The differential expression analysis revealed notable changes in genes primarily associated with immune response and inflammation in mice exposed to the CIE model. For instance, the gene Cd74, which exhibited the highest overexpression (FC = 6.446) in the CIE group, is known for its role in activated microglia and proinflammatory signaling (Hwang et al., 2017). Several genes related to MHCII (H2-Aa, H2-Eb1, H2-Ab1, H2-Q6, H2-D1, H2-Q7, H2-Q4, H2-K1) were also upregulated. MHCII expression is known to increase in activated microglia in response to proinflammatory cytokines (Panek and Benveniste, 1995). Similarly, numerous genes implicated in the immune responses, pro-inflammatory signaling, macrophage function and leukocyte trafficking, including Igtp, Ifi209, Iigp1, Ifi27l2a, the Gbp family, Bst2, Siglec1, Usp18, Nlrc5, Dusp2, Clec4a2, Fut7, Treml2, and Cd274 or Pd-L1, showed increased expression (Ford and McVicar, 2009; Wei et al., 2013; Schneider et al., 2017; Basters et al., 2018; Siddiqui et al., 2019; Tiwari et al., 2019; Mishra et al., 2020; Nasu et al., 2020; Haque et al., 2021; Zhang et al., 2021).

Among the downregulated genes, Glra1 and Kcne2 stand out. Glra1 encodes the α1 subunit of the glycine receptor (GlyR), which is broadly expressed in the CNS and has been suggested to play a role in modulating the reward system in response to EtOH exposure (Förstera et al., 2017; Gallegos et al., 2019). On the other hand, KCNE2 encodes a transmembrane protein that is part of various potassium channels. This protein is prominently expressed on the apical membrane of the choroid plexus, where it influences the composition of cerebrospinal fluid and indirectly affects neuronal excitability (Abbott et al., 2014).

The top 20 enriched genes, identified through an ontology-based gene enrichment analysis, were represented in a dot plot. This analysis confirmed that the CIE group shows neuroinflammation and an altered immune response compared with the Control group (Figure 3D).

### 3.3 CIE model increases plasma and brain concentrations of KYN and KYN/TRP ratio

The CIE model produced alterations in anxiety and memory along with an increase in transcripts related to the immune response. The KYN pathway is known to be activated under inflammatory conditions and has also been associated with elevated anxiety and cognitive impairments. Based on this information, we explored the impact of the CIE model on the activation of the KYN pathway, a possible link between EtOH consumption, neuroinflammation and behavioral alterations (Jiang et al., 2020; dos Santos et al., 2021). To this end, we examined the levels of KYN, TRP, and 5-HT, and calculated the KYN/TRP and 5-HT/TRP ratios as indicators of TDO + IDO and TPH enzyme activities, respectively, 24 h post-vapor exposure.

**FIGURE 4:**
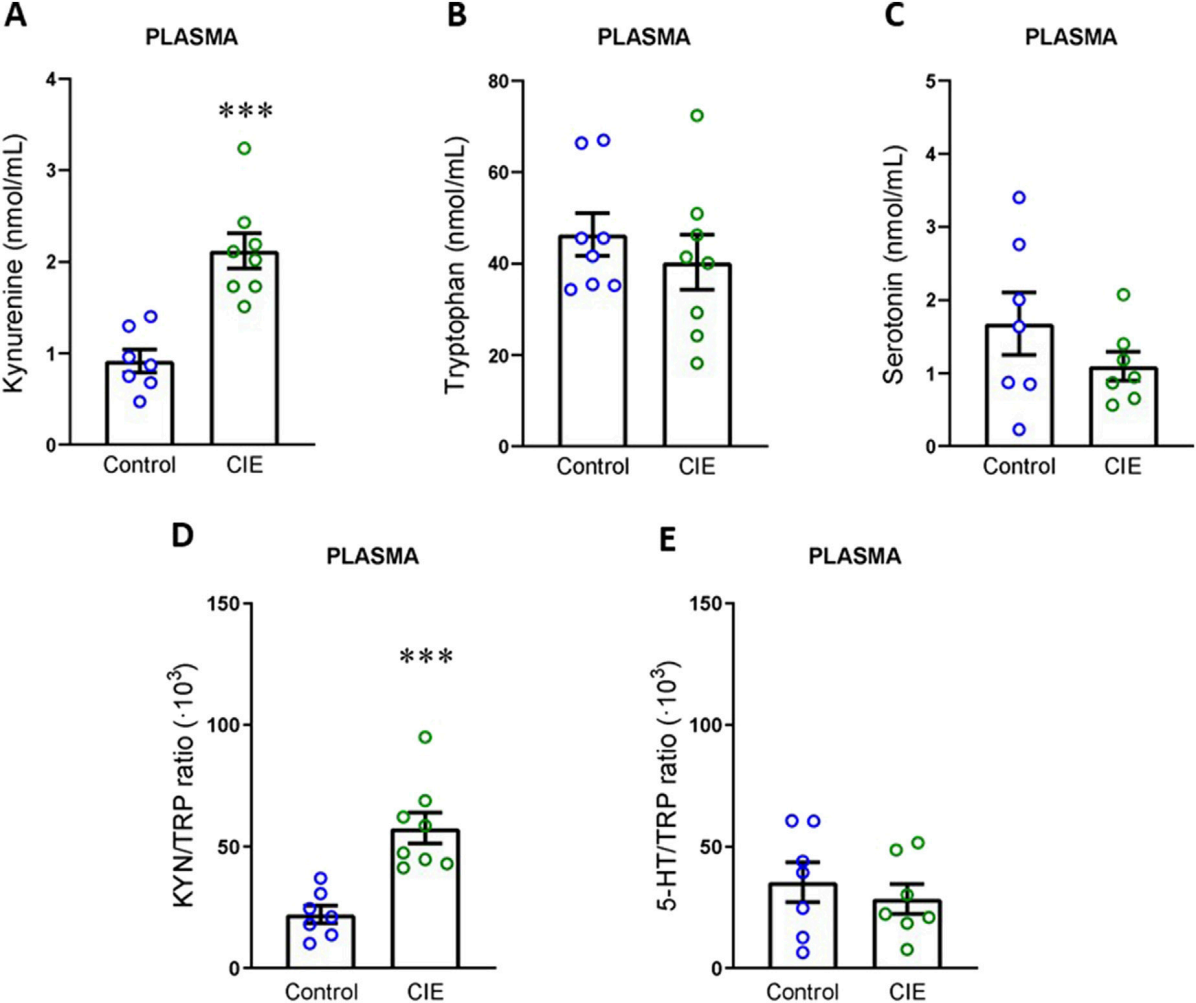
Impact of CIE model on kynurenine pathway in plasma. **(A)** Kynurenine concentration. **(B)** Tryptophan concentration. **(C)** Serotonin concentration. **(D)** KYN/TRP ratio. **(E)** TRP/5-HT ratio All the determinations were performed 24 h following the final exposure to EtOH vapors. Results are presented as mean ± SEM, n: Control = 7 and CIE = 8 for A and D, Control and CIE = 8 for B, Control and CIE = 7 for C and E Statistically different from Control: ***p < 0.001, using *t*-test for A, C, D, E, and *U* test for B.

**FIGURE 5:**
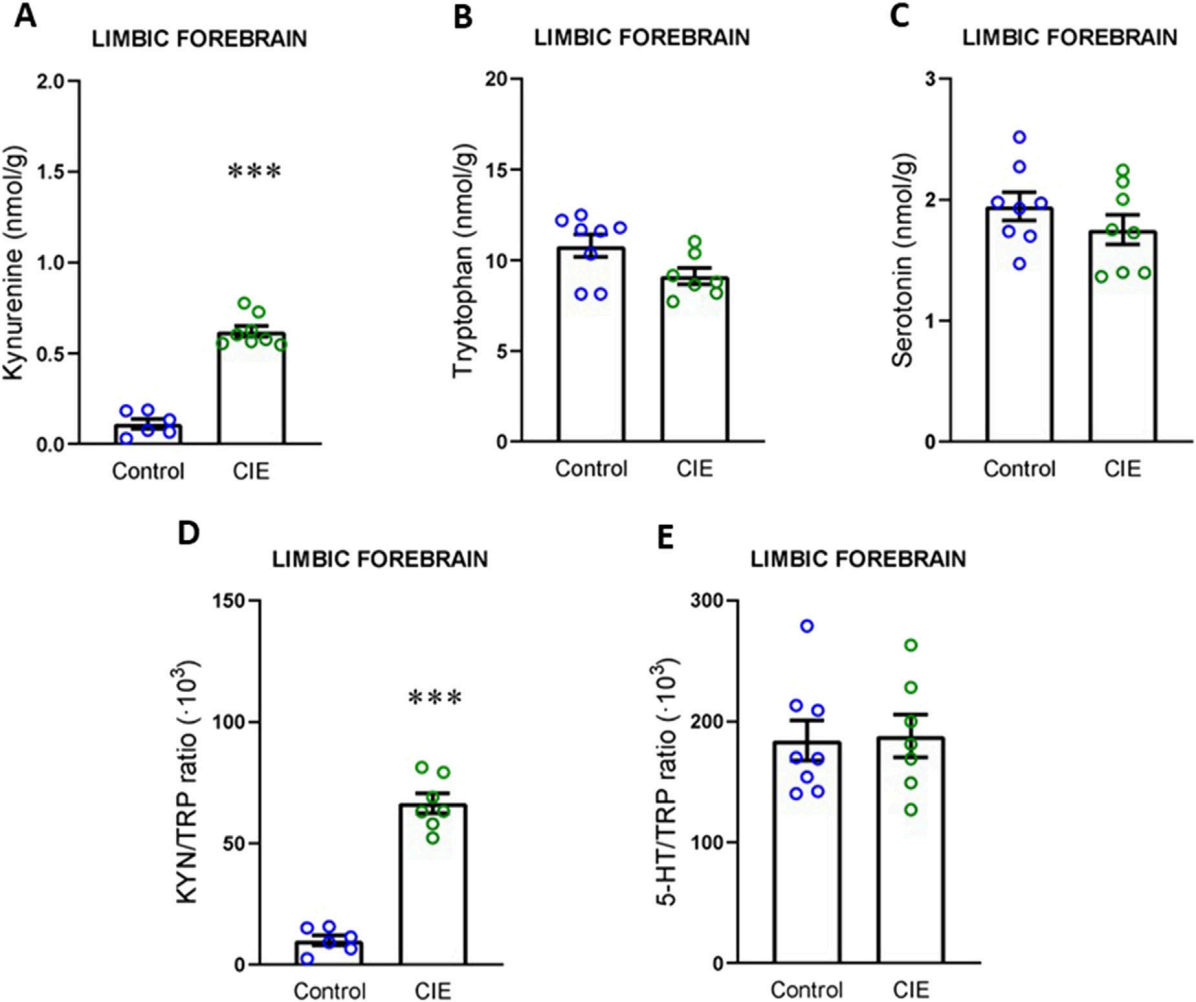
Impact of CIE model on kynurenine pathway in limbic forebrain. **(A)** Kynurenine concentration. **(B)** Tryptophan concentration. **(C)** Serotonin concentration. **(D)** KYN/TRP ratio. **(E)** TRP/5-HT ratio All the determinations were performed 24 h following the final exposure to EtOH vapors. Results are presented as mean ± SEM, n: Control = 6 and CIE = 8 for A, Control = 8 and CIE = 7 for B and E, Control and CIE = 8 for C, Control = 6 and CIE = 7 for D Statistically different from Control: ***p < 0.001, using *t*-test for A, C, D, E, and *U* test for B.

First, we analyzed the activation of the KYN pathway in plasma. Our data demonstrated that KYN levels were elevated in plasma (Figure 4A; t (13) = 5.09, p = 0.0002), with no changes in TRP (Figure 4B; U = 26, p = 0.574, exact) or 5-HT levels (Figure 4C; t (12) = 1.24, p = 0.239). As a result of this increase in circulating KYN levels, the KYN/TRP ratio (Figure 4D; t (13) = 4.65, p = 0.0005) was elevated, while the 5-HT/TRP ratio (Figure 4E; t (12) = 0.67, p = 0.514) remained unchanged. Next, we focused on the limbic forebrain, which includes the NAc, where RNAseq analysis has been performed. In this area, we observed an increase in KYN levels (Figure 5A; t (12) = 12.13, p < 0.0001), with no changes in TRP (Figure 5B; U = 14, p = 0.121, exact) or 5-HT levels (Figure 5C; t (14) = 1.13, p = 0.276). Similarly, the KYN/TRP ratio was elevated in the CIE group (Figure 5D; t (11) = 11.80, p < 0.0001), while the 5-HT/TRP ratio remained unchanged (Figure 5E; t (13) = 0.15, p = 0.883).

**FIGURE 6:**
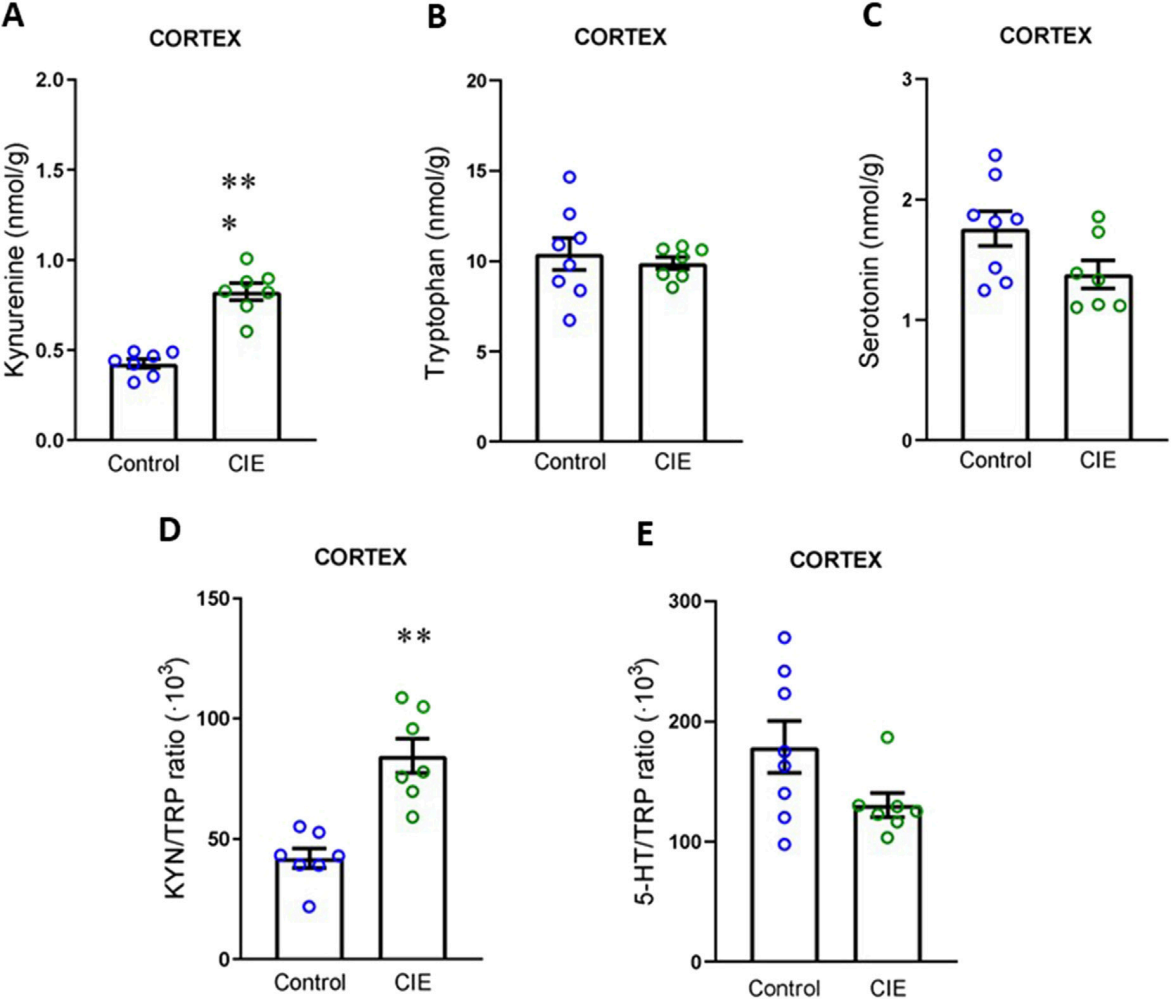
Impact of CIE model on kynurenine pathway in cortex. **(A)** Kynurenine concentration. **(B)** Tryptophan concentration. **(C)** Serotonin concentration. **(D)** KYN/TRP ratio. **(E)** TRP/5-HT ratio All the determinations were performed 24 h following the final exposure to EtOH vapors. Results are presented as mean ± SEM, n: Control and CIE = 7 for A and D, Control = 8 and CIE = 7 for B, C and E Statistically different from Control: **p < 0.01, ***p < 0.001, using *t*-test for A, B, C, E, and *U* test for E.

**FIGURE 7:**
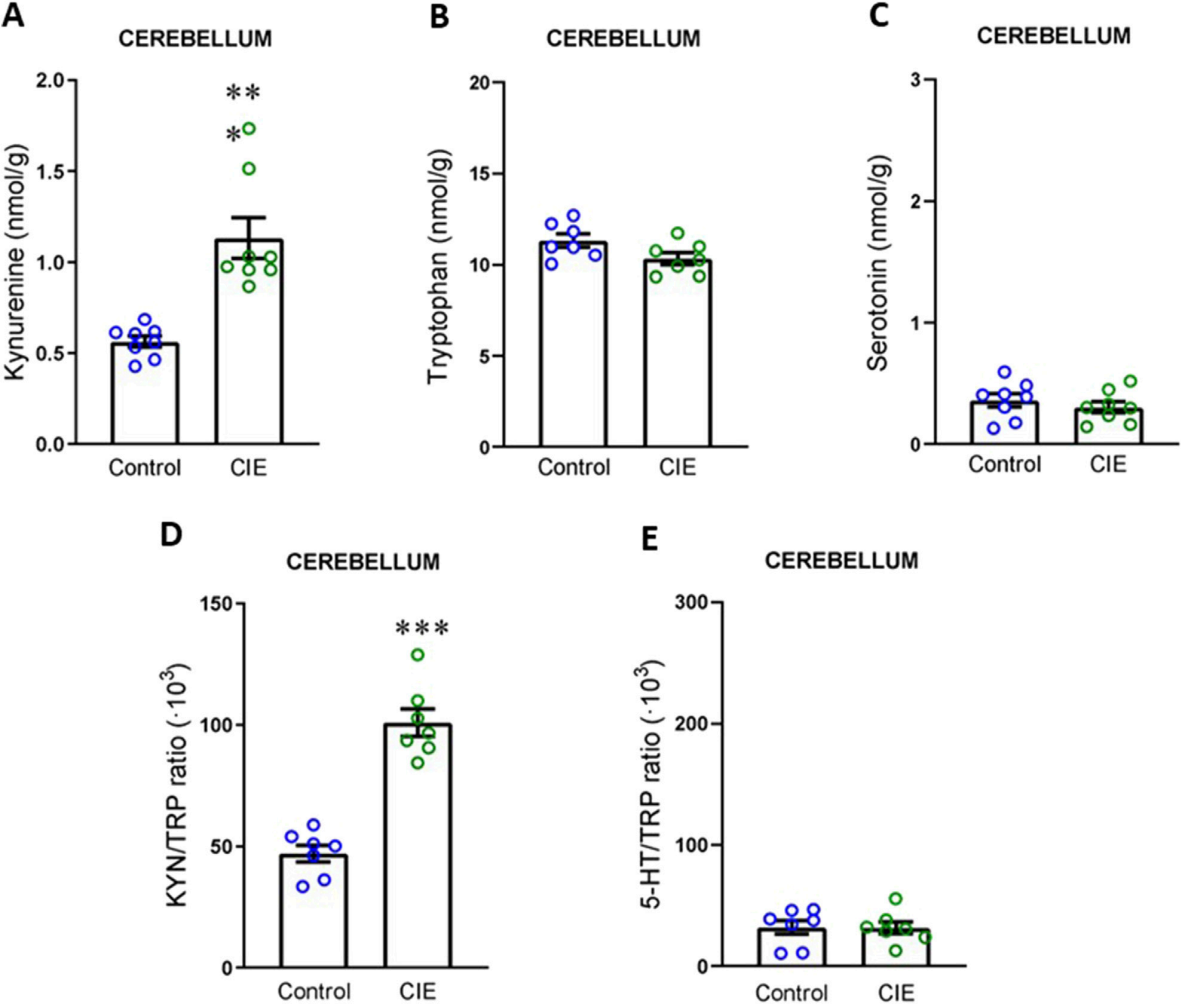
Impact of CIE model on kynurenine pathway in cerebellum. **(A)** Kynurenine concentration. **(B)** Tryptophan concentration. **(C)** Serotonin concentration. **(D)** KYN/TRP ratio. **(E)** TRP/5-HT ratio All the determinations were performed 24 h following the final exposure to EtOH vapors. Results are presented as mean ± SEM, n: Control and CIE = 8 for A and C, Control and CIE = 7 for B, D, and E Statistically different from Control: ***p < 0.001, using *t*-test for B, C, D, E, and *U* test for A.

Finally, given that the cortex and cerebellum have been implicated in previously described alterations (Miquel et al., 2015; Koob, 2014), we aimed to investigate whether changes in KYN levels were also observable in these areas. Our results showed that KYN levels were elevated in cortex (Figure 6A; t (12) = 7.35, p < 0.0001), with no changes in TRP (Figure 6B; t (13) = 0.49, p = 0.632) or 5-HT levels (Figure 6C; t (13) = 2.02, p = 0.065). The KYN/TRP ratio was increased (Figure 6D; t (12) = 5.18, p = 0.0002), while the 5-HT/TRP ratio remained unchanged (Figure 6E; U = 15, p = 0.152, exact). In the cerebellum (Figures 7A–E), we observed the same pattern as in the previous regions, with increased KYN levels (U = 0, p = 0.0002, exact) and KYN/TRP ratio (t (12) = 8.14, p < 0.0001), and no changes in TRP (t (12) = 2.01, p = 0.068), 5-HT (t (14) = 0.81, p = 0.432) or 5-HT/TRP ratio (t (12) = 0.052, p = 0.960).

To determine whether the increase in KYN levels persisted over time, we analyzed the levels of KYN, TRP, and 5-HT in the limbic forebrain 72 h post-withdrawal and found no significant differences in any of the metabolites analyzed: KYN (Supplementary Figure S1D; t (11) = 1.621, p = 0.133), TRP (Supplementary Figure S1E; t (12) = 0.2480, p = 0.808), or 5-HT (Supplementary Figure S1F; t (12) = 0.2459, p = 0.810).

## 4 Discussion

In this study, we propose that EtOH dependence in mice triggers activation of the KYN pathway through immune system activation, accompanied by affective and cognitive alterations. We employed the CIE model of dependence and withdrawal, which produces an escalation in EtOH consumption after three cycles of exposure to ethanol vapor, resulting in a pattern of excessive EtOH intake (Lopez and Becker, 2005; Lopez et al., 2017; Gil de Biedma-Elduayen et al., 2022) and withdrawal symptoms (Riegle et al., 2014; Patel et al., 2021), mimicking the transition from voluntary consumption to dependence.

CIE-exposed mice exhibited anxiety-like symptoms and memory impairment 24 h post-EtOH exposure, as assessed by the EPM and NOR, respectively. Prior studies have reported heightened anxiety indicators following 24 h of abstinence in animal models of chronic EtOH exposure (van Skike et al., 2015; Jiang et al., 2020; Gakare et al., 2022). Similarly, numerous studies demonstrate that moderate and chronic EtOH administration in rodents leads to impairments in memory and other cognitive functions, even after withdrawal, assessed through the NOR and other behavioral tests (Stragier et al., 2015; Kato et al., 2016; Contreras et al., 2019). It is important to note that some of these behavioral effects may be mediated by acetaldehyde. Although the role of acetaldehyde in the effects of alcohol is still not fully understood, a growing body of evidence indicates that acetaldehyde administration induces a range of behavioral effects, suggesting that some of alcohol’s actions may be mediated, at least in part, by its primary metabolite (Quertemont and Didone, 2006).

Conversely, no alterations in anhedonia were observed. Although this contrasts with prior findings (Giménez-Gómez et al., 2019; Hartmann et al., 2020), it is worth noting that none of those studies used the same EtOH exposure protocol employed here. Other groups have also failed to detect changes in sucrose preference in rodents following chronic EtOH exposure (Nelson et al., 2018; Olney et al., 2018), and some suggest that depressive signs only manifest after a prolonged period of abstinence (2 weeks–2 months) (Stevenson et al., 2009; Holleran et al., 2016). In line with our results, dos Santos et al., proposed that short-term ethanol withdrawal produced anxiety-like behaviors, while long-term withdrawal favored depressive-like behaviors (dos Santos et al., 2021). Chronic ethanol consumption has been associated with depression and mood disorders (McHugh and Weiss, 2019); however, these are multifactorial conditions that are very complex to model and assess preclinically (Gururajan et al., 2019). In this study, we focused on anhedonia, a core component of mood disorders, using the sucrose preference test. Although this is a well-established paradigm for evaluating anhedonia (Berrio et al., 2024), it should be noted that it addresses only one aspect of mood-related behavior. Therefore, it would be premature to draw definitive conclusions about depressive-like symptoms in CIE mice based solely on this measure. Future studies should incorporate additional behavioral tests to assess anhedonia and explore other relevant dimensions of mood disorders.

We analyzed the transcriptome of the NAc in mice subjected to the CIE model, 24 h after EtOH vapor exposure. Although this is the main cerebral nucleus implicated in the regulation of the positive reinforcing effects of EtOH, it has been also implicated in other functions such as stress, anxiety, and cognition (Castro and Bruchas, 2019). Moreover, it has been established that dopamine modulation can restore limbic memory deficits and synaptic plasticity in the NAc following alcohol withdrawal indicating a key role of this area not only in the reinforcing effects of EtOH but also cognitive dysfunctions (Cannizzaro et al., 2019).

Gene ontology analysis revealed that differentially expressed genes in CIE-exposed mice were primarily involved in immune response processes. Among the overexpressed genes, we identified Cd74 and several MHCII-related genes (H2-Aa, H2-Eb1, H2-Ab1, H2-Q6, H2-D1, H2-Q7, H2-Q4, H2-K1), known as markers for activated microglia, especially during proinflammatory processes (Panek and Benveniste, 1995; Hwang et al., 2017). MHCII expression increases in response to interferon gamma (IFN-γ) (Panek and Benveniste, 1995). Additionally, other immune response genes regulated by IFN-γ and IFN-α/β, such as Igtp, Ifi209, Iigp1, Ifi27l2a, various Gbp family genes, Bst2, Siglec1, Usp18, and Nlrc5, were also upregulated (Basters et al., 2018; Siddiqui et al., 2019; Tiwari et al., 2019; Haque et al., 2021; Zhang et al., 2021). These results suggest that CIE exposure may alter signaling through IFN, consistent with prior publications (Lawrimore et al., 2018; Kapoor et al., 2019).

Finally, we identified other upregulated genes associated with inflammation, leukocyte trafficking, proinflammatory cytokine production, and the immune response, such as Dusp2, Clec4a2, Fut7, Treml2, and Cd274 or PD-L1 (Ford and McVicar, 2009; Wei et al., 2013; Schneider et al., 2017; Mishra et al., 2020; Nasu et al., 2020). Of these, only Cd274 has been previously associated with EtOH consumption (Mishra et al., 2020).

Our findings align with previous studies employing transcriptomic analysis after various EtOH exposure models in various brain regions. These investigations consistently identified changes in the expression of genes involved in immune response activation and inflammation following acute EtOH administration (Zhang et al., 2015), binge drinking models in rats (McClintick et al., 2018), chronic consumption in mice (Osterndorff-Kahanek et al., 2013; Mccarthy et al., 2018), and in the CIE model, specifically 8 h post-EtOH exposure (Osterndorff-Kahanek et al., 2015). Furthermore, analyses in patients with AUD have shown both upregulation and downregulation of genes involved in immunity and inflammation in the brain (Liu et al., 2005; McClintick et al., 2013; Kapoor et al., 2019), with “signaling through IFN” presenting the highest enrichment (Kapoor et al., 2019). We found several downregulated genes, such as Glra1 and Kcne2, but none of them were implicated in the immune system response. Taken together, these data, including ours, indicate immune system activation following EtOH exposure. However, a limitation of transcriptomic studies is that altered gene expression does not always translate to changes at the protein level, as protein levels are often more conserved and regulated post-transcriptionally (Perl et al., 2017). Therefore, further experiments are crucial to confirm whether the differentially expressed genes are indeed translated into proteins.

In brief, the transcriptome analysis of the NAc suggests that exposure to the CIE model triggers activation of the neuroimmune system in EtOH-exposed animals, as indicated in several studies (Coleman and Crews, 2018; Erickson et al., 2019). Over recent years, the KYN pathway has gained attention as an immune regulator (O’Farrell and Harkin, 2017). Although TDO is the primary enzyme responsible for TRP degradation, under inflammatory conditions, IDO may become more crucial. Different proinflammatory cytokines, including IFN and TNFα, can increase IDO activity, elevating KYN levels (O’Farrell and Harkin, 2017; Kim and Jeon, 2018). We decided to measure KYN levels and the KYN/TRP ratio in both plasma and limbic forebrain to determine the status of the KYN pathway both peripherally and centrally.

Contrary to the transcriptomic study, for the HPLC measurements we used the limbic forebrain instead of the NAc. This decision was based on the small size of the NAc, preventing sufficient tissue collection for HPLC analysis without significantly increasing the experiment size, in agreement with the ethical animal use guidelines. The limbic forebrain was considered an acceptable alternative as it includes the NAc and other reward system structures. These findings correspond with our previous research using a chronic consumption model that does not induce dependence (Giménez-Gómez et al., 2018). In that model, we observed increased KYN levels in plasma and the limbic forebrain immediately after ceasing consumption, but not 24 h later. This suggests that the CIE model induces longer-lasting KYN pathway changes, potentially linked to brain alterations during the transition to dependence. Previous research has suggested that EtOH administration can activate TDO and IDO enzymes, leading to KYN accumulation (Badawy et al., 2009; Jiang et al., 2020; dos Santos et al., 2021), as observed in this study. Elevated KYN levels during abstinence in individuals with AUD have been detected, associated with abstinence duration and AUD severity (Gleissenthall et al., 2014; Neupane et al., 2015; Vidal et al., 2020), implying a potential role of the KYN pathway in the heightening stress sensitivity during abstinence in individuals with AUD (Morales-Puerto et al., 2021).

The relationship between KYN levels in the blood and brain is well-documented, with peripheral changes generally reflected in the brain (Schwarcz et al., 2012), as observed in the present study. However, previous research suggests that under certain conditions, discrepancies can arise in the KYN pathway between the brain and serum, and even among different brain regions (Parrott et al., 2016; Giménez-Gómez et al., 2021). Based on this information, we decided to analyze KYN, TRP and 5-HT in cortex and cerebellum, two regions that have been also associated with alcohol dependence (Miquel et al., 2015; Koob, 2014). In both regions, we found patterns consistent with those observed in plasma and the limbic forebrain, with elevated KYN levels and KYN/TRP ratio. Altogether, these results indicate that CIE increases KYN levels and KYN/TRP ratio in both plasma and brain.

In recent decades, inflammation and anxiety have been increasingly associated, as elevated pro-inflammatory cytokines are often observed in individuals with anxiety, and immune activation has been shown to induce anxiety-like behaviors in both animals and humans (Peirce and Alviña, 2019). This connection is well established in the context of the KYN pathway (Kim and Jeon, 2018). Inflammatory conditions can activate IDO, leading to elevated KYN levels that have anxiogenic effects in animal models (Lapin et al., 1996) and correlate with anxiety severity in humans (Orlikov and Ryzov, 1991; Orlikov et al., 1994). Moreover, recent research links the anxiety observed after 24 h of abstinence to IDO activation in dependent mice (Jiang et al., 2020), aligning with our findings. This evidence suggests that the anxiety changes observed after the CIE model may also be related to elevated KYN levels, reinforcing the idea that inflammation-driven IDO activity contributes to anxiety. Furthermore, it will be of interest to explore downstream metabolites of the KYN pathway, such as QUIN and KYNA, which have been previously implicated in anxiety-related behaviors, in the context of ethanol exposure. Similar to KYN, QUIN has been reported to exert anxiogenic effects, whereas KYNA may act as an anxiolytic (Lapin et al., 1996; Lapin et al., 1996). Additionally, the interaction between the KYN pathway and other key neuroimmune signaling molecules remains an underexplored but promising area of investigation. For example, potential interactions with toll-like receptors (Crews et al., 2017) or neuropeptide Y (Brancato et al., 2023) merit further investigation. Altogether, these lines of research could reinforce the concept that ethanol exposure disrupts multiple neuromodulatory systems, ultimately contributing to behavioral pathology.

Research similarly highlights a strong link between inflammation and memory impairment. Chronic inflammation, characterized by elevated cytokines and other inflammatory markers, has been associated with cognitive decline over time (Liu et al., 2020; Schmidt-Morgenroth et al., 2023). While the relationship between inflammation, KYN, and memory impairment is not fully established, existing evidence suggests that alterations in the KYN pathway contribute to cognitive deficits. Specifically, an elevated KYN/TRP ratio has been associated to poorer associative memory and language performance in humans (Solvang et al., 2019; Chirico et al., 2020), which aligns with our results. Further research is needed to explore the connection between neuroinflammation, KYN pathway activation, and cognitive impairment, particularly in the context of AUD.

Finally, we replicated the behavioral assessments (EPM and NOR) and measured KYN, TRP, and 5-HT, 72 h after EtOH exposure to characterize the temporal dynamics of the observed changes. Interestingly, all effects had reversed by this time point, indicating that the behavioral and metabolic alterations are transient. This finding is consistent with previous publications from our group demonstrating that EtOH-induced changes in the KYN pathway are temporary (Giménez-Gómez et al., 2019) and support the hypothesis that the behavioral impairments observed at 24 h may be associated with a transient accumulation of KYN in the brain. However, it remains unclear whether longer or repeated EtOH exposure (i.e., more than four CIE cycles) could result in more persistent or irreversible changes.

It should be noted that our study was conducted exclusively in male mice. Future studies will be necessary to examine whether the effects observed also occur in females and if so to what extent. This is particularly important given that females are more vulnerable than males to the neurotoxic and negative consequences of chronic alcohol consumption (Cruz et al., 2023), potentially leading to more extensive effects that may not have been captured in the present study. Along the same lines, it is also relevant to consider ethanol exposure during critical periods of development. Previous studies have shown that binge-like ethanol exposure from the preconception period can induce significant alterations in the affective phenotype of the offspring (Brancato et al., 2018). These findings highlight the need to determine whether the effects observed in our study could be exacerbated or more far-reaching if exposure occurs during sensitive developmental windows.

To summarize, this study reveals that neuroimmune activation following chronic EtOH exposure may trigger IDO enzyme activation, leading to elevated KYN concentrations and potentially contributing to the anxiety and memory alterations observed in animals exposed to the CIE model. However, while our findings suggest a relationship between these changes and are valuable at pointing to future lines of research, the exact mechanism remains unknow, which represents a limitation of the study. Future research will be needed to clarify the causal role of IDO in mediating the observed behavioral and molecular alterations. Approaches such as pharmacological inhibition using 1-methyl-tryptophan (1-MT), a well-established IDO inhibitor, or the use of genetic models such as IDO1 knockout mice, could help determine whether IDO activity is directly responsible for the CIE-induced behavioral changes, immune gene expression, and kynurenine pathway activation.

## Data Availability

The data presented in the study are deposited in the Docta Complutense repository, accession number https://hdl.handle.net/20.500.14352/121767.

## Abbreviations

2BC, Two-bottle choice limited access; 5-HT, Serotonin; AUD, Alcohol Use Disorder; CIE, Chronic Intermittent ethanol; CNS, central nervous system; DI, Discrimination index; EPM, Elevated plus maze; EtOH, ethanol; IDO, Indolamine 2,3-dioxygenase; IFN, Interferon; KYN, Kynurenine; NAc, Nucleus accumbens; NOR, Novel object recognition test; OFT, Open field test; TRP, Tryptophan; TDO, Tryptophan 2,3-dioxygenase; TPH, Tryptophan hydroxylase.
