# Supplementary material for: The kynurenine pathway as a potential link between ethanol-induced behavioral alterations and neuroinflammation
Source: Front Pharmacol. 2025 Jul 7;16:1628527. doi: 10.3389/fphar.2025.1628527 (PMC12277286; doi:10.3389/fphar.2025.1628527)
Supplement: Supplementary file 1 [file DataSheet1.docx]

**Supplementary Table 1**

| **FIGURE 1.** | | | | |  |  |
| --- | --- | --- | --- | --- | --- | --- |
| **FIGURE 1B. ETHANOL CONSUMPTION** | | | | | **Baseline** | **Cycle 3** |
|  | | | W | | 0,9176 | 0,8813 |
|  | | | P value | | 0,4110 | 0,1936 |
|  | | | Passed normality test (alpha=0.05)? | | Yes | Yes |
| **FIGURE 1C. PREFERENCE** | | | | | **Baseline** | **Cycle 3** |
|  | | | W | | 0,8796 | 0,9094 |
|  | | | P value | | 0,1865 | 0,3498 |
|  | | | Passed normality test (alpha=0.05)? | | Yes | Yes |
| **FIGURE 1D. WATER CONSUMPTION** | | | | | **Baseline** | **Cycle 3** |
|  | | | W | | 0,8990 | 0,9694 |
|  | | | P value | | 0,2832 | 0,8929 |
|  | | | Passed normality test (alpha=0.05)? | | Yes | Yes |
| **FIGURE 1E. WEIGHT** | | | | | **Control** | **CIE** |
|  | | | W | | 0,9404 | 0,9123 |
|  | | | P value | | 0,5863 | 0,3321 |
|  | | | Passed normality test (alpha=0.05)? | | Yes | Yes |
|  | | |  | |  |  |
| **FIGURE 2.** | | | | |  |  |
| **FIGURE 2A. DISTANCE TRAVELED (OFT)** | | | | | **Control** | **CIE** |
|  | | | W | | 0,9545 | 0,9685 |
|  | | | P value | | 0,7707 | 0,8860 |
|  | | | Passed normality test (alpha=0.05)? | | Yes | Yes |
| **FIGURE 2B. AVERAGE SPEED (OFT)** | | | | | **Control** | **CIE** |
|  | | | W | | 0,9498 | 0,9689 |
|  | | | P value | | 0,7281 | 0,8895 |
|  | | | Passed normality test (alpha=0.05)? | | Yes | Yes |
| **FIGURE 2C. SUCROSE PREFERENCE** | | | | | **Control** | **CIE** |
|  | | | W | | 0,8226 | 0,9628 |
|  | | | P value | | 0,0497 | 0,8365 |
|  | | | Passed normality test (alpha=0.05)? | | No | Yes |
| **FIGURE 2D. TIME IN OPEN ARMS (EPM)** | | | | | **Control** | **CIE** |
|  | | | W | | 0,8857 | 0,8799 |
|  | | | P value | | 0,2964 | 0,2262 |
|  | | | Passed normality test (alpha=0.05)? | | Yes | Yes |
| **FIGURE 2E. ENTRIES INTO OPEN ARMS (EPM)** | | | | | **Control** | **CIE** |
|  | | | W | | 0,8841 | 0,7891 |
|  | | | P value | | 0,2454 | 0,0319 |
|  | | | Passed normality test (alpha=0.05)? | | Yes | No |
| **FIGURE 2F. DISCRIMINATION INDEX (NOR)** | | | | | **Control** | **CIE** |
|  | | | W | | 0,9590 | 0,9480 |
|  | | | P value | | 0,8101 | 0,7115 |
|  | | | Passed normality test (alpha=0.05)? | | Yes | Yes |
|  | | |  | |  |  |
| **FIGURE 4. PLASMA** | | | | |  |  |
| **FIGURE 4A. KYNURENINE** | | | | | **Control** | **CIE** |
|  | | | W | | 0,9511 | 0,8976 |
|  | | | P value | | 0,7394 | 0,2750 |
|  | | | Passed normality test (alpha=0.05)? | | Yes | Yes |
| **Figure 4B. TRYPTOPHAN** | | | | | **Control** | **CIE** |
|  | | | W | | 0,8069 | 0,9572 |
|  | | | P value | | 0,0339 | 0,7835 |
|  | | | Passed normality test (alpha=0.05)? | | No | Yes |
| **FIGURE 4C. SEROTONIN** | | | | | **Control** | **CIE** |
|  | | | W | | 0,9595 | 0,9116 |
|  | | | P value | | 0,8145 | 0,4067 |
|  | | | Passed normality test (alpha=0.05)? | | Yes | Yes |
| **FIGURE 4D. KYN/TRP** | | | | | **Control** | **CIE** |
|  | | | W | | 0,9759 | 0,8556 |
|  | | | P value | | 0,9376 | 0,1085 |
|  | | | Passed normality test (alpha=0.05)? | | Yes | Yes |
| **FIGURE 4E. TRP/5-HT** | | | | | **Control** | **CIE** |
|  | | | W | | 0,9204 | 0,9093 |
|  | | | P value | | 0,4723 | 0,3907 |
|  | | | Passed normality test (alpha=0.05)? | | Yes | Yes |
|  | | |  | |  |  |
| **FIGURE 5. LIMBIC FOREBRAIN** | | | | |  |  |
| **FIGURE 5A. KYNURENINE** | | | | | **Control** | **CIE** |
|  | | | W | | 0,9052 | 0,8323 |
|  | | | P value | | 0,4056 | 0,0628 |
|  | | | Passed normality test (alpha=0.05)? | | Yes | Yes |
| **Figure 5B. TRYPTOPHAN** | | | | | **Control** | **CIE** |
|  | | | W | | 0,8070 | 0,9352 |
|  | | | P value | | 0,0340 | 0,5958 |
|  | | | Passed normality test (alpha=0.05)? | | No | Yes |
| **FIGURE 5C. SEROTONIN** | | | | | **Control** | **CIE** |
|  | | | W | | 0,9658 | 0,8899 |
|  | | | P value | | 0,8630 | 0,2335 |
|  | | | Passed normality test (alpha=0.05)? | | Yes | Yes |
| **FIGURE 5D. KYN/TRP** | | | | | **Control** | **CIE** |
|  | | | W | | 0,9477 | 0,9385 |
|  | | | P value | | 0,7217 | 0,6256 |
|  | | | Passed normality test (alpha=0.05)? | | Yes | Yes |
| **FIGURE 5E. TRP/5-HT** | | | | | **Control** | **CIE** |
|  | | | W | | 0,8713 | 0,9816 |
|  | | | P value | | 0,1552 | 0,9671 |
|  | | | Passed normality test (alpha=0.05)? | | Yes | Yes |
| **FIGURE 6. CORTEX** | | | | |  |  |
| **FIGURE 6A. KYNURENINE** | | | | | **Control** | **CIE** |
|  | | | W | | 0,8945 | 0,9673 |
|  | | | P value | | 0,2987 | 0,8781 |
|  | | | Passed normality test (alpha=0.05)? | | Yes | Yes |
| **Figure 6B. TRYPTOPHAN** | | | | | **Control** | **CIE** |
|  | | | W | | 0,9894 | 0,8809 |
|  | | | P value | | 0,9941 | 0,2303 |
|  | | | Passed normality test (alpha=0.05)? | | Yes | Yes |
| **FIGURE 6C. SEROTONIN** | | | | | **Control** | **CIE** |
|  | | | W | | 0,9302 | 0,8517 |
|  | | | P value | | 0,5177 | 0,1272 |
|  | | | Passed normality test (alpha=0.05)? | | Yes | Yes |
| **FIGURE 6D. KYN/TRP** | | | | | **Control** | **CIE** |
|  | | | W | | 0,9090 | 0,9330 |
|  | | | P value | | 0,3892 | 0,5768 |
|  | | | Passed normality test (alpha=0.05)? | | Yes | Yes |
| **FIGURE 6E. TRP/5-HT** | | | | | **Control** | **CIE** |
|  | | | W | | 0,9579 | 0,7783 |
|  | | | P value | | 0,7895 | 0,0248 |
|  | | | Passed normality test (alpha=0.05)? | | Yes | No |
| **FIGURE 7. CEREBELLUM** | | | | |  |  |
| **FIGURE 7A. KYNURENINE** | | | | | **Control** | **CIE** |
|  | | | W | | 0,9537 | 0,7487 |
|  | | | P value | | 0,7483 | 0,0079 |
|  | | | Passed normality test (alpha=0.05)? | | Yes | No |
| **FIGURE 7B. TRYPTOPHAN** | | | | | **Control** | **CIE** |
|  | | | W | | 0,9578 | 0,9477 |
|  | | | P value | | 0,7994 | 0,7086 |
|  | | | Passed normality test (alpha=0.05)? | | Yes | Yes |
| **FIGURE 7C. SEROTONIN** | | | | | **Control** | **CIE** |
|  | | | W | | 0,9558 | 0,9457 |
|  | | | P value | | 0,7691 | 0,6675 |
|  | | | Passed normality test (alpha=0.05)? | | Yes | Yes |
| **FIGURE 7D. KYN/TRP** | | | | | **Control** | **CIE** |
|  | | | W | | 0,9299 | 0,9212 |
|  | | | P value | | 0,5502 | 0,4786 |
|  | | | Passed normality test (alpha=0.05)? | | Yes | Yes |
| **FIGURE 7E. TRP/5-HT** | | | | | **Control** | **CIE** |
|  | | | W | | 0,8169 | 0,9523 |
|  | | | P value | | 0,0600 | 0,7506 |
|  | | | Passed normality test (alpha=0.05)? | | Yes | Yes |
|  | | |  | |  |  |
| **FIGURE SUPPLEMENTARY 2.** | | | | |  |  |
| **FIGURE SUP. 2A. TIME IN OPEN ARMS (EPM)** | | | | | **Control** | **CIE** |
|  | | | W | | 0,8848 | 0,9031 |
|  | | | P value | | 0,2093 | 0,3080 |
|  | | | Passed normality test (alpha=0.05)? | | Yes | Yes |
| **FIGURE SUP. 2B. ENTRIES INTO OPEN ARMS (EPM)** | | | | | **Control** | **CIE** |
|  | | W | | | 0,8615 | 0,9255 |
|  | | P value | | | 0,1244 | 0,4761 |
|  | | Passed normality test (alpha=0.05)? | | | Yes | Yes |
| **FIGURE SUP. 2C. DISCRIMINATION INDEX (NOR)** | | | | | **Control** | **CIE** |
|  | W | | | | 0,9469 | 0,9240 |
|  | P value | | | | 0,6797 | 0,5348 |
|  | Passed normality test (alpha=0.05)? | | | | Yes | Yes |
| **FIGURE SUP. 2D. KYNURENINE LIMBIC FOREBRAIN** | | | | | **Control** | **CIE** |
|  | | | | W | 0,9273 | 0,8059 |
|  | | | | P value | 0,4918 | 0,0904 |
|  | | | | Passed normality test (alpha=0.05)? | Yes | Yes |
| **FIGURE SUP. 2E. TRYPTOPHAN LIMBIC FOREBRAIN** | | | | | **Control** | **CIE** |
|  | | | | W | 0,8807 | 0,9202 |
|  | | | | P value | 0,1913 | 0,5070 |
|  | | | | Passed normality test (alpha=0.05)? | Yes | Yes |
| **FIGURE SUP. 2F. SEROTONINE LIMBIC FOREBRAIN** | | | | | **Control** | **CIE** |
|  | | | | W | 0,9108 | 0,9550 |
|  | | | | P value | 0,3599 | 0,7803 |
|  | | | | Passed normality test (alpha=0.05)? | Yes | Yes |


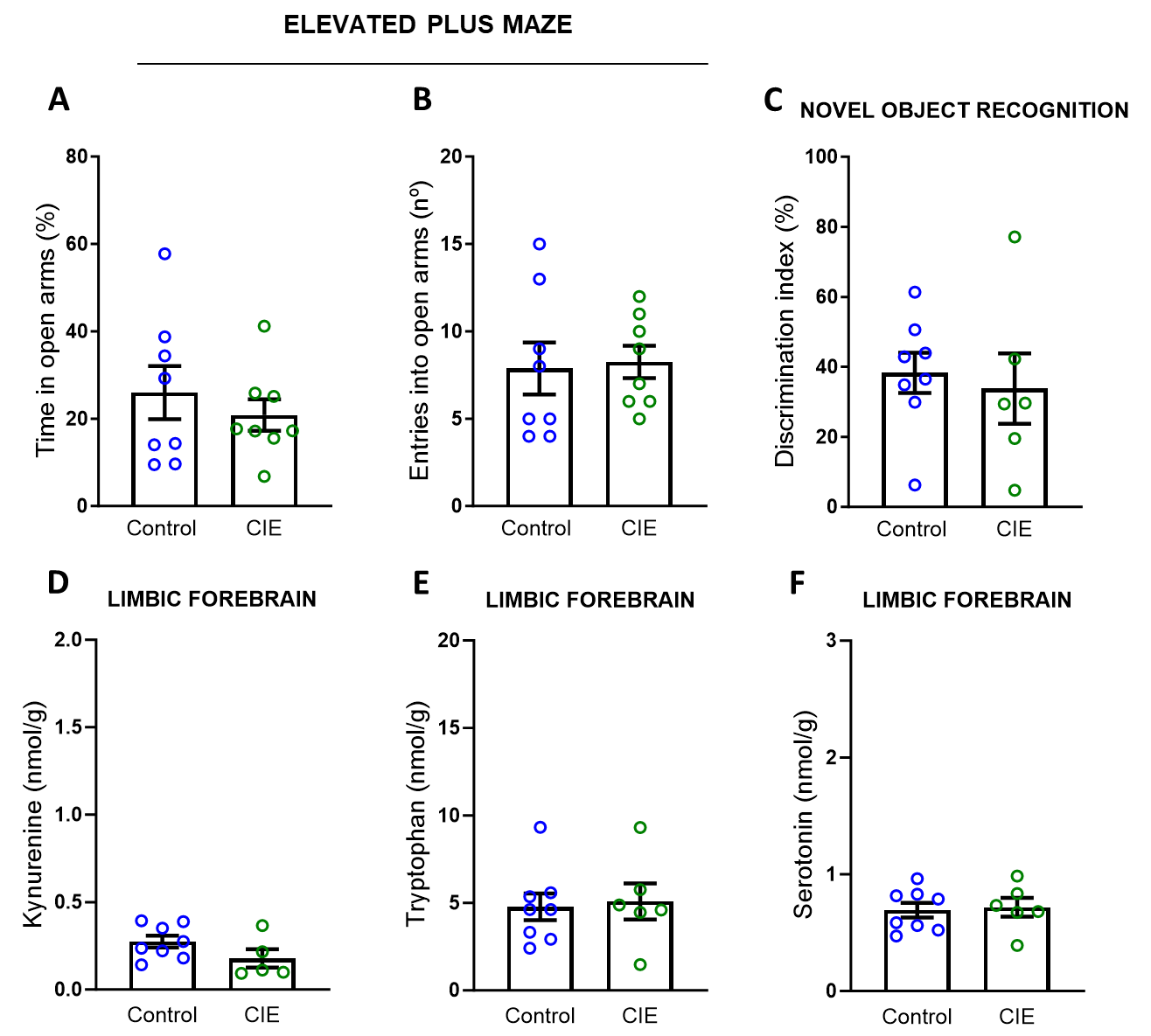


Supplementary Figure 1. Effect of the CIE model on behavioral and cognitive parameters and in the kynurenine pathway, 72 hours after the last EtOH-exposure. **A.** Time spent in open arms (%) and **B**, number of entries into open arms in the elevated plus maze test. **C**. Discrimination index (%) in the novel object recognition test. **D.** Kynurenine concentration, **E.** Tryptophan concentration and **F**. Serotonin concentration in the limbic forebrain. All the tests were conducted 72 hours after the final exposure to EtOH vapors. Results are presented as mean ± SEM; n: Control = 8 and CIE = 8 for A and B, 6 for C, E and F, 5 for D.


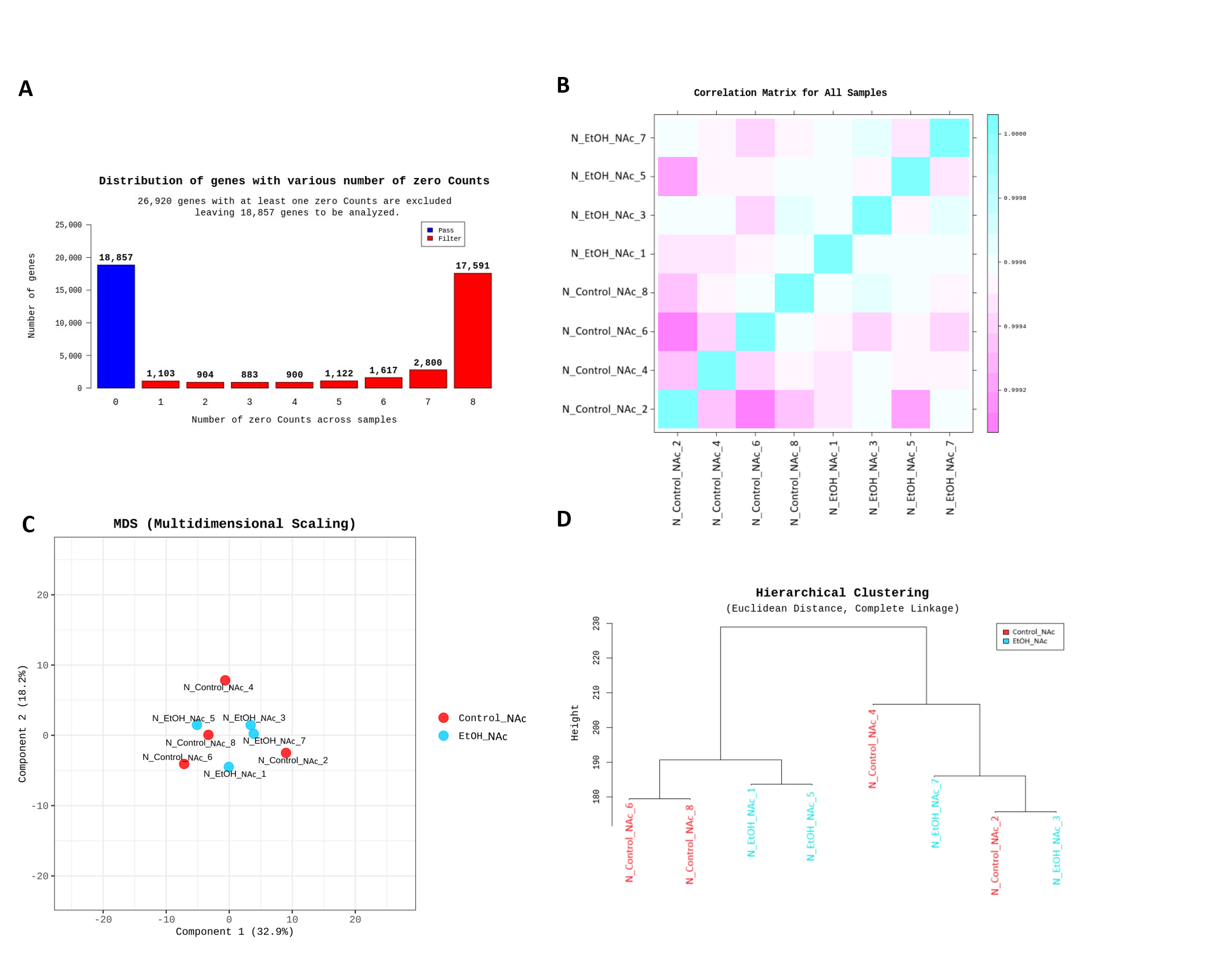


**Supplementary Figure 2.** Quality control of sequenced samples from the NAc of mice exposed to the CIE paradigm. **A**. Gene distribution with multiple zero read counts. 26,920 genes with at least one read count equal to 0 were excluded, leaving 18,857 valid genes for statistical analysis. **B**. Correlation matrix among all samples, calculated using the Pearson coefficient. For the range: -1 ≤ r ≤ 1, the closer the value is to 1, the more similar the samples are. The color in this graph represents the association between the different samples. **C**. 2D multidimensional scaling diagram. This graph distributes the samples according to the variance in expression among them. Variance is represented by the principal components found on the X-axis (PC1: 32.9%) and the Y-axis (PC2: 18.2%). Before representation, a logarithmic transformation of the values of different samples was performed to account for small differences among them. **D**. Dendrogram of the analyzed samples. The set of samples is hierarchically represented using Euclidean distance. In this dendrogram, samples are grouped without a logarithmic transformation of their transcript expression. Significant differences among the samples are considered during grouping.

**Supplementary Table 2.** List of differentially expressed genes.

| GEN | DESCRIPTION | GENE BIOTYPE | FOLD CHANGE |
| --- | --- | --- | --- |
| Cd74 | CD74 antigen (invariant polypeptide of major histocompatibility complex, class II antigen-associated) | protein_coding | **6,446** |
| Gdf1 | growth differentiation factor 1 | protein_coding | **4,011** |
| Igtp | interferon gamma induced GTPase | protein_coding | **3,956** |
| H2-Aa | histocompatibility 2, class II antigen A, alpha | protein_coding | **3,575** |
| H2-Eb1 | histocompatibility 2, class II antigen E beta | protein_coding | **3,483** |
| B430319G15Rik | RIKEN cDNA B430319G15 gene | lncRNA | **3,355** |
| H2-Ab1 | histocompatibility 2, class II antigen A, beta 1 | protein_coding | **3,284** |
| Psmb9 | proteasome (prosome, macropain) subunit, beta type 9 (large multifunctional peptidase 2) | protein_coding | **3,190** |
| Ifi209 | interferon activated gene 209 | protein_coding | **3,129** |
| Gm40798 | predicted gene, 40798 | lncRNA | **3,099** |
| Heatr4 | HEAT repeat containing 4 | protein_coding | **3,006** |
| Iigp1 | interferon inducible GTPase 1 | protein_coding | **2,925** |
| H2-Q6 | histocompatibility 2, Q region locus 6 | protein_coding | **2,890** |
| Gbp10 | guanylate-binding protein 10 | protein_coding | **2,856** |
| Dusp2 | dual specificity phosphatase 2 | protein_coding | **2,771** |
| 2810454H06Rik | RIKEN cDNA 2810454H06 gene | lncRNA | **2,749** |
| Nlrc5 | NLR family, CARD domain containing 5 | protein_coding | **2,721** |
| Fut7 | fucosyltransferase 7 | protein_coding | **2,677** |
| Gbp6 | guanylate binding protein 6 | protein_coding | **2,652** |
| Gm31796 | predicted gene, 31796 | lncRNA | **2,642** |
| LOC115488173 | uncharacterized LOC115488173 | lncRNA | **2,603** |
| Psmb8 | proteasome (prosome, macropain) subunit, beta type 8 (large multifunctional peptidase 7) | protein_coding | **2,550** |
| Gm4951 | predicted gene 4951 | protein_coding | **2,538** |
| Oasl2 | 2'-5' oligoadenylate synthetase-like 2 | protein_coding | **2,536** |
| Clec4a2 | C-type lectin domain family 4, member a2 | protein_coding | **2,520** |
| H2-D1 | histocompatibility 2, D region locus 1 | protein_coding | **2,487** |
| Wt1 | Wilms tumor 1 homolog | protein_coding | **2,477** |
| Gm10336 | predicted gene 10336 | lncRNA | **2,461** |
| Slc22a18 | solute carrier family 22 (organic cation transporter), member 18 | protein_coding | **2,457** |
| H2-Q7 | histocompatibility 2, Q region locus 7 | protein_coding | **2,445** |
| Tgtp2 | T cell specific GTPase 2 | protein_coding | **2,392** |
| Ifi27l2a | interferon, alpha-inducible protein 27 like 2A | protein_coding | **2,383** |
| Tgtp1 | T cell specific GTPase 1 | protein_coding | **2,381** |
| Gm6548 | predicted gene 6548 | Transcribed pseudogene | **2,323** |
| H2-Q4 | histocompatibility 2, Q region locus 4 | protein_coding | **2,312** |
| Gm35170 | predicted gene, 35170, transcript variant X3 | lncRNA | **2,306** |
| H2-K1 | histocompatibility 2, K1, K region | protein_coding | **2,292** |
| C330020E22Rik | RIKEN cDNA C330020E22 gene | lncRNA | **2,276** |
| Bst2 | bone marrow stromal cell antigen 2 | protein_coding | **2,201** |
| Fhl4 | four and a half LIM domains 4 | protein_coding | **2,194** |
| Gbp2 | guanylate binding protein 2 | protein_coding | **2,186** |
| Treml2 | triggering receptor expressed on myeloid cells-like 2 | protein_coding | **2,160** |
| Tap1 | transporter 1, ATP-binding cassette, sub-family B (MDR/TAP) | protein_coding | **2,157** |
| Irgm2 | immunity-related GTPase family M member 2 | protein_coding | **2,135** |
| Siglec1 | sialic acid binding Ig-like lectin 1, sialoadhesin | protein_coding | **2,106** |
| Usp18 | ubiquitin specific peptidase 18 | protein_coding | **2,083** |
| LOC102632463 | uncharacterized LOC102632463 | lncRNA | **2,077** |
| Cd274 | CD274 antigen | protein_coding | **2,076** |
| Atf3 | activating transcription factor 3 | protein_coding | **2,060** |
| Irgm1 | immunity-related GTPase family M member 1 | protein_coding | **2,025** |
| Ddx60 | DEAD (Asp-Glu-Ala-Asp) box polypeptide 60 | protein_coding | **2,016** |
| Oas1a | 2'-5' oligoadenylate synthetase 1A | protein_coding | **2,014** |
| Usp43 | ubiquitin specific peptidase 43 | protein_coding | **-2,006** |
| Gm40640 | predicted gene, 40640 | lncRNA | **-2,053** |
| Gm6475 | predicted gene 6475 | pseudogene | **-2,059** |
| Wdr86 | WD repeat domain 86 | protein_coding | **-2,088** |
| Gm31852 | predicted gene, 31852, transcript variant X2 | lncRNA | **-2,097** |
| Gm46767 | predicted gene, 46767 | lncRNA | **-2,114** |
| Gm19345 | predicted gene, 19345 | protein_coding | **-2,118** |
| A930009A15Rik | RIKEN cDNA A930009A15 gene | protein_coding | **-2,120** |
| Gm31075 | predicted gene, 31075, transcript variant X3 | lncRNA | **-2,120** |
| Gm30085 | predicted gene, 30085 | lncRNA | **-2,158** |
| Ecrg4 | ECRG4 augurin precursor | protein_coding | **-2,190** |
| Gm34941 | predicted gene, 34941 | lncRNA | **-2,211** |
| Gm41287 | predicted gene, 41287 | lncRNA | **-2,260** |
| Gm46100 | predicted gene, 46100, transcript variant X1 | protein_coding | **-2,260** |
| Kcnh8 | potassium voltage-gated channel, subfamily H (eag-related), member 8 | protein_coding | **-2,284** |
| Gpr50 | G-protein-coupled receptor 50 | protein_coding | **-2,297** |
| Lemd1 | LEM domain containing 1 | protein_coding | **-2,313** |
| Plin5 | perilipin 5 | protein_coding | **-2,361** |
| Gm32082 | predicted gene, 32082 | lncRNA | **-2,595** |
| Gm34502 | predicted gene, 34502, transcript variant X4 | lncRNA | **-2,671** |
| Aqp1 | aquaporin 1 | protein_coding | **-2,693** |
| Glra1 | glycine receptor, alpha 1 subunit | protein_coding | **-2,758** |
| Gm44504 | predicted readthrough transcript (NMD candidate), 44504 | protein_coding | **-2,766** |
| Gm4833 | predicted gene 4833 | pseudogene | **-2,873** |
| Pif1 | PIF1 5'-to-3' DNA helicase | protein_coding | **-2,892** |
| Kcne2 | potassium voltage-gated channel, Isk-related subfamily, gene 2 | protein_coding | **-3,290** |
| F5 | coagulation factor V | protein_coding | **-3,712** |
| Ttr | transthyretin | protein_coding | **-6,091** |
| Rsc1a1 | regulatory solute carrier protein, family 1, member 1 | protein_coding | **-9,628** |
